# New evidence for complex climate change in MIS 11 from Hoxne, Suffolk, UK

**DOI:** 10.1016/j.quascirev.2008.01.003

**Published:** 2008-04

**Authors:** Nick Ashton, Simon G. Lewis, Simon A. Parfitt, Kirsty E.H. Penkman, G. Russell Coope

**Affiliations:** a Department of Prehistory and Europe, British Museum, Franks House, 56 Orsman Road, London N1 5QJ, UK; b Department of Geography, Queen Mary, University of London, Mile End Road, London E1 4NS, UK; c Department of Palaeontology, Natural History Museum, Cromwell Road, London SW7 5BD, UK; d Institute of Archaeology, University College London, 31-34 Gordon Square, London WC1H 0PY, UK; e BioArCh, Departments of Biology, Archaeology and Chemistry, University of York, Heslington, York YO10 5DD, UK; f Tigh-na-Cleirich, Foss, nr Pitlochry, Perthshire PH16 5NQ, UK

## Abstract

The climatic signal of Marine Isotope Stage (MIS) 11 is well-documented in marine and ice-sheet isotopic records and is known to comprise at least two major warm episodes with an intervening cool phase. Terrestrial records of MIS 11, though of high resolution, are often fragmentary and their chronology is poorly constrained. However, some notable exceptions include sequences from the maar lakes in France and Tenaghi Philippon in Greece. In the UK, the Hoxnian Interglacial has been considered to correlate with MIS 11. New investigations at Hoxne (Suffolk) provide an opportunity to re-evaluate the terrestrial record of MIS 11. At Hoxne, the type Hoxnian Interglacial sediments are overlain by a post-Hoxnian cold-temperate sequence. The interglacial sediments and the later temperate phase are separated by the so-called ‘Arctic Bed’ from which cold-climate macroscopic plant and beetle remains have been recovered. The later temperate phase was deposited during an episode of boreal woodland and is associated with the artefacts, a diverse vertebrate fauna and molluscs. New amino acid geochronological data and biostratigraphical considerations suggest that the post-Hoxnian sequence correlates with late substages of MIS 11. The paper further investigates the correlation of the sequence at Hoxne with the palynological sequences found elsewhere in Europe and adjacent offshore areas.

## 1 Introduction

In recent years, the complexity and structure of Marine Isotope Stage (MIS) 11 has been a focus of research, in part driven by the similarity of orbitally forced insolation changes during MIS 11 and the Holocene (Oppo et al., 1998; Droxler et al., 2003; Loutre and Berger, 2003; Ruddiman, 2005; Wu et al., 2007). MIS 11 is therefore important as an analogue for current and future climate scenarios. An important aspect of this work is how global temperature changes affect terrestrial biota, which can be addressed through the correlation of marine and terrestrial records (Tzedakis et al., 1997; Desprat et al., 2005; Wu et al., 2007). It has become increasingly clear that there is a much more complex relationship between the often fragmented terrestrial record and the marine and ice records. The complexity of MIS 11 has now been shown through marine and ice-sheet isotope records (e.g. Bassinot et al., 1994; EPICA Community Members, 2004) and long palynological records from marine cores (Desprat et al., 2005). These all indicate a sharp warming at ca 425 ka with what appears to be a relatively stable climate through to ca 390 ka. Thereafter, the records are characterised by a series of warm–cold oscillations until ca 360 ka with the onset of more extreme cold.

**Fig. 1:**
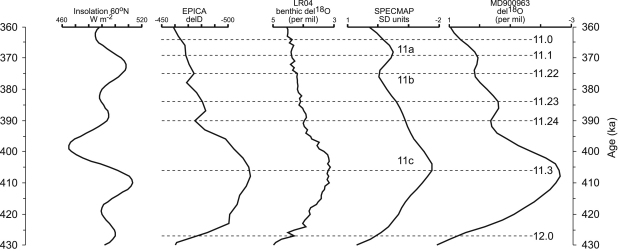
Structure and sub-division of MIS 11 as shown by isotopic records from ocean and ice cores. Isotope substages after Tzedakis et al. (2001), isotopic events after Bassinot et al. (1994). Sources: Insolation, Berger and Loutre (1991); EPICA deuterium record, EPICA community members (2004); LR04 benthic stack, Lisiecki and Raymo (2005); SPECMAP stack, Imbrie et al. (1984); MD900963, Bassinot et al. (1994).

Two conventions have been established for the naming of isotopic and therefore climatic fluctuations within numbered isotope stages. MIS 11 has been divided into substages 11c, 11b and 11a (e.g. Tzedakis et al., 2001). However, in other records (e.g. MD900963, Bassinot et al., 1994) a more complex pattern can be seen with additional warm–cold oscillation (Fig. 1). Therefore, an alternative system identifies negative and positive isotopic events, which are numbered using a decimal system (Imbrie et al., 1984; Bassinot et al., 1994; Desprat et al., 2005). This has the advantage of allowing additional isotopic events to be incorporated. These two conventions are different because the first denotes periods of time, whereas the second identifies specific isotopic events, and therefore the terminology should not be used interchangeably. An additional short-lived warm episode has been recognised in some records (e.g. Prokopenko et al., 2001) and referred to as 11e, but this is not prominent in either SPECMAP or MD900963. The structure and terminology for MIS 11 is used in this paper are shown in Fig. 1.

Only recently has it been possible to recognise these complex changes in the terrestrial record, particularly in southern Europe where pollen sequences have enabled correlation with marine isotope substages (Reille and de Beaulieu, 1995; Tzedakis et al., 1997; de Beaulieu et al., 2001; Desprat et al., 2005; Tzedakis et al., 2006). In Britain, such sequences have not been found, and most palynological records are of relatively short duration (cf. Thomas, 2001). Although there is now widespread agreement that the Anglian glaciation correlates with MIS 12 and the Hoxnian Interglacial broadly with MIS 11 (Bridgland, 1994; Bowen, 1999; Rowe et al., 1999; Grün and Schwarcz, 2000; Preece and Penkman, 2005; Preece et al., 2007), individual substages of MIS 11 have not been convincingly identified (but see Schreve (2001a, b)). The climatic record of the Hoxnian has been largely based on palynology (West, 1956; Turner, 1970), but it is still not clear whether the Hoxnian encompasses the whole of MIS 11 or just one substage. One site that has the potential to provide information of this kind is the site of Hoxne, Suffolk, UK (TM176767), where recent fieldwork has shown a more complex sequence of climate fluctuations that may be attributable to marine isotope substages.

The sequence at Hoxne forms the stratotype of the Hoxnian Interglacial (Mitchell et al., 1973) which, based on the analysis of West (1956) of the lacustrine sediments, spans pollen zones HoI-III. However, the stratigraphy also includes an important series of sediments that post-date this lacustrine sequence. These are of particular importance because they contain abundant palaeoenvironmental evidence and also a rich Palaeolithic archaeological record (Singer et al., 1993). Despite the long history of research at Hoxne, it remains unclear how the primary context human industries relate to the environmental record and the correlation of this part of the succession with the marine isotope sequence is uncertain (Bowen et al., 1989; Gladfelter et al., 1993; Turner and West 1994; Ashton et al., 1995; West and Gibbard, 1995; Grün and Schwarcz 2000; Schreve 2001a, b). The current research has focussed on these issues as part of a wider investigation of human presence during MIS 11 under the auspices of the Ancient Human Occupation of Britain Project (Stringer and AHOB Project Members, 2003; Ashton et al., 2006).

**Fig. 2:**
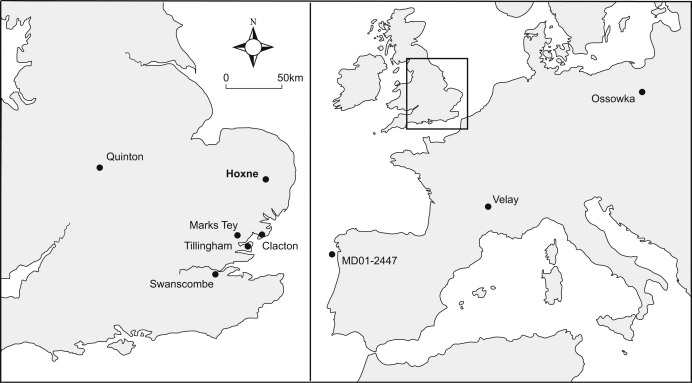
Location of sites mentioned in the text.

**Fig. 3:**
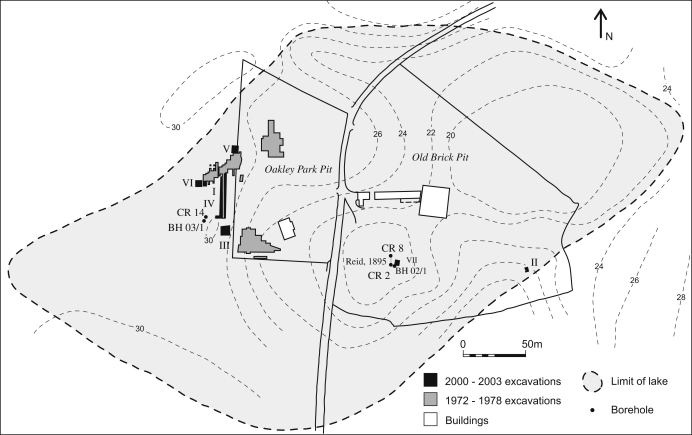
Site location and plan. The basin contours (mOD) and limits of the lake are based on West (1956).

Since the discovery at Hoxne of Lower Palaeolithic handaxes by Frere (1800), the site has been the subject of several investigations, providing often radical re-interpretations of previous work. This work has focussed on two pits, either side of the Hoxne to Eye road (Figs. 2 and 3). The Old Brick Pit to the east of the road was the subject of the earliest investigations, and these were supplemented by work in the Oakley Park Pit when this was first opened in the mid-19th century. The two pits were opened for gravel and clay extraction from the infillings of a basin that is now located on the interfluve between the Goldbrook stream and River Dove, which flow into the River Waveney. The current research has involved investigation of archive material together with cutting and sampling of key sections (between 2000 and 2003; Ashton et al., 2003) and has enabled a re-evaluation of the palaeoenvironmental and archaeological evidence from the site.

## 2 Previous interpretations

**Fig. 4:**
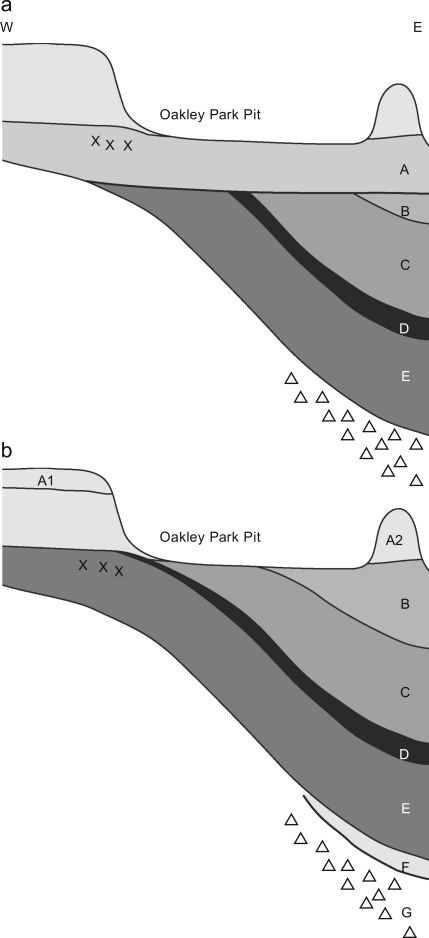
Stratigraphical interpretation of (a) Reid (Evans et al., 1896) in comparison to that of (b) West (1956). Modified from West and McBurney (1954, Fig. 2) and from West (1956, Fig. 15).

**Fig. 5:**
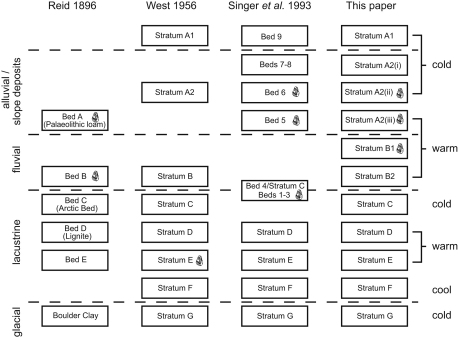
The interpretations of the stratigraphy of Reid (Evans et al., 1896), West (1956), Singer et al. (1993) and current work. The handaxe symbols show the contexts in which artefacts were thought to be located.

The basis for much of our understanding of the site comes from the work of Clement Reid (Evans et al., 1896). He provided detailed and clear descriptions from boreholes and open sections of all the major sediment units and undertook the first palaeobotanical investigations at the site. Reid's work demonstrated that a basin, formed in the surface of the ‘boulder clay’ (till), was infilled with interglacial lacustrine sediment (Bed E). Drying out of the lake was indicated by the formation of peat (Bed D), prior to the re-establishment of the lake under cold conditions (‘Arctic Bed’: Bed C). These lacustrine sediments were overlain by a fluvial, shelly, gravel (Bed B), and the artefact-bearing sediment or ‘Palaeolithic Loam’ (Bed A). The latter extended beyond the confines of the basin (Figs. 4 and 5).

In the 1950s, detailed fieldwork was undertaken by West (1956), who also examined the palynology of the lake sediments. West argued for important modifications to the stratigraphy offered by Reid. Other than the addition of a cold lacustrine sediment (Stratum F) above the till (now Stratum G), he argued that the lower part of Reid's Bed A had been misinterpreted and was in fact decalcified Bed (now Stratum) E. This implied that the human occupation of the site was associated with the lacustrine sediments of the newly defined Hoxnian Interglacial, rather than in the later sediments at the site (Figs. 4 and 5). No archaeology was discovered in Stratum E during West's work, other than two flakes from sections 40 and 100 on the west of the Oakley Park Pit (West, 1956; Fig. 3). However, it is clear from re-examination of the section drawings and the heights of the objects in comparison to those found in the 1970s and during the recent excavation that they were actually recovered from sediments overlying Stratum D. West (1956) defined pollen zones at Hoxne, which were subsequently modified following work on the more complete sequence at Marks Tey, Essex. The Hoxne lake sediments (Strata E–D) were assigned to pollen zones HoI through to HoIIIa, with HoIIIb and HoIV being absent from the sequence (Turner, 1970).

Major archaeological excavations by the University of Chicago in the 1970s (Singer et al., 1993) in the Oakley Park Pit and in the field to the west of this pit (Fig. 3), provided the first properly excavated artefact assemblages from the site and led to further re-interpretation. It was argued that there were two significant phases of human occupation. The first or ‘Lower Industry’ consisted of primary context handaxes, cores and knapping debitage in association with a temperate faunal assemblage, and occurred in a single horizon towards the base of Stratum C. A new bed nomenclature was introduced for a sequence of fluvial, alluvial and solifluction sediments (Beds 1–9) that lie above Stratum D (Gladfelter, 1993). These had formerly been attributed to Strata B and A. The key units were a chalky gravel (Bed 4), which was overlain by a fine-grained alluvial sediment (Bed 5) and a further gravel (Bed 6). The ‘Upper Industry’, consisting of pointed handaxes, flake tools, cores and debitage in variable condition, was recovered from the top of Bed 5 and in secondary context in the lower part of Bed 6. The ‘Lower Industry’ was associated with a temperate vertebrate fauna. However, this conflicts with the palaeobotanical evidence of Reid (in Evans et al., 1896) and West (1956) who attributed Stratum C (‘Arctic Bed’) to a cold stage.

The dating of the site and its correlation with the marine isotope record was also unclear from this work (Gladfelter et al., 1993; Turner and West, 1994). Amino acid D/L ratios on *Valvata* shells from Stratum E suggested a correlation of the lacustrine sequence with MIS 9 (Bowen et al., 1989), with the implication that either there is a major hiatus between the Lowestoft Till (MIS 12) and the lake beds, or that the glacial sequence dates to MIS 10. Dating of the overlying Lower Industry (interpreted as lying in Stratum C) was assessed through thermoluminescence dating on two burnt flints, which yielded a mean date of 210±20 ka, suggesting an MIS 7 age. However, the dosimeter readings (taken several years after the excavation) were from different locations and also demonstrated considerable variation across the site. This suggested that there were likely to be considerable errors in the TL age estimates (Bowman, 1993). Initial ESR dates on enamel from two horse (*Equus ferus*) teeth also associated with the Lower Industry gave an average age of 319±38 ka, suggesting that Stratum C was attributable to MIS 9 (Schwarcz and Grün, 1993). However, subsequent remodelling of the data has suggested an MIS 11 age with dates of 404±33/42 and 437±38 ka (Grün and Schwarcz, 2000). Finally, assessment of the mammalian fauna that is associated with the Lower Industry and from Bed 4 has been argued to show marked similarities with that from Swanscombe (Stuart et al., 1993), and both faunal assemblages have been attributed to the first post-Anglian warm stage and assigned to the Swanscombe Mammal Assemblage Zone (MAZ) by Schreve (2001a, b).

## 3 Stratigraphic re-interpretation

A revised stratigraphy is proposed, based on the curent re-investigation of the site that focussed on the relationship between Stratum C and the sediments containing the Lower and Upper Industries (Fig. 5; Table 1). This has resolved the confusion in Singer et al. (1993) in their varied interpretations of the relationship between Stratum C and Bed 4 (see Bridgland, 1994; Turner and West, 1994). The stratigraphic interpretations and nomenclature of Singer et al. (1993) can now be reconciled with that of West (1956). This paper uses the Reid/West nomenclature, with some modifications based on the current research (Table 1).

**Fig. 6:**
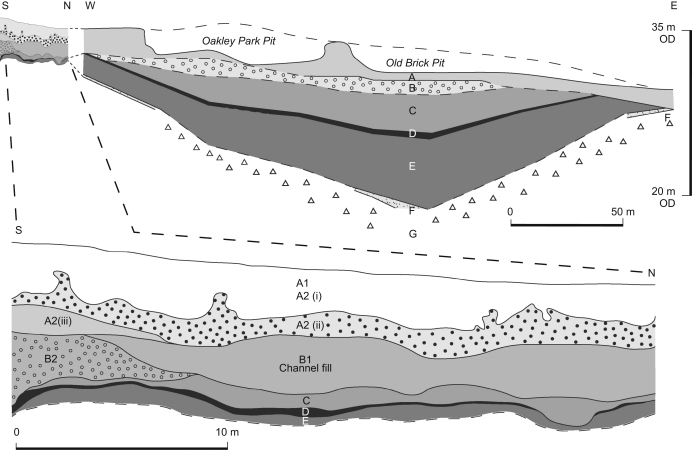
Schematic cross-section through the Hoxne lake basin (after West, 1956) with a detailed section through Area IV.

Fig. 3 shows the location of the recently excavated sections (Areas I–VII) and boreholes. The most significant information comes from Areas III and IV. The latter was a narrow trench, previously excavated in 1978 that was re-opened and widened to allow detailed investigation and sampling. The trench linked the locations of the Lower and the Upper Industries in the field to the west of the Oakley Park Pit (Figs. 3 and 6). This section is critical to understanding the relationship of the Upper and Lower Industries to Stratum C and Beds 4–6.

Stratum E was exposed at the base, overlain by a near-continuous horizon of Stratum D, which reached a maximum thickness of 35 cm at the southern end, but thinned to <1 cm black stained clay at the northern end (Fig. 6). Overlying this was a thin horizon of Stratum C, 50-cm thick over most of the exposure, but cut out at the southern end by fine sand and chalky gravel of Stratum B.

Above this, a concave-up erosional lower bounding surface is incised into Strata C and B, forming a broad (>30 m), shallow (ca 2 m) channel feature, infilled with bedded sands, silts and clays. These are interpreted as a lateral accretion facies and indicate lateral movement to the north. The orientation of bone long axes and their distribution with that of the artefacts also suggest a fluvial deposit and indicate a NE–SW orientation of the channel. This channel feature was not recognised by previous workers and the sediment was thought to be part of the lacustrine sequence. It is here assigned to Stratum B because it is a fluvial deposit and is referred to as Stratum B1. The underlying chalky gravel is therefore assigned to Stratum B2 (Fig. 5).

Stratum A was sub-divided into A1 and A2 by West (1956). As a result of the current work, Stratum A2 is further sub-divided into A2(i–iii). Stratum A2(iii), a sandy clay unit, is interpreted as alluvium. Above this, a coarse flint gravel with a sandy clay matrix (Stratum A2(ii)) and a series of laminated sands and silts (A2(i)) are capped by gravely sands (Stratum A1). Post-depositional disturbance and downslope movement have affected the uppermost part of the succession.

## 4 Palaeoenvironmental context for human occupation at Hoxne

The sedimentary succession at the site contains palaeoenvironmental data indicating a fluctuating climate. The depositional environment, vegetational and faunal character, and thermal conditions can be considered for each stratum in turn.

### 4.1 Stratum G

The ‘boulder clay’ of Reid was assigned to the Lowestoft Till by West (1956) and represents widespread glaciation of eastern England by a British-based ice-sheet depositing the characteristic chalk and flint-rich tills of the Lowestoft Formation (Perrin et al., 1979; Bowen, 1999; Clark et al., 2004). This glaciogenic unit is attributable to the Anglian Stage (MIS 12).

### 4.2 Stratum F

This lacustrine clay lies at the bottom of the basin and contains pollen and beetles (West, 1956; Coope, 1993). The beetle remains indicate a rapid amelioration of climate to near interglacial conditions during the late Anglian.

### 4.3 Stratum E

These lake sediments form the major filling of the basin and contain a pollen sequence that has been attributed to pollen zones HoI—HoIIc of the Hoxnian Interglacial. The pollen indicates development of fully temperate deciduous woodland (West, 1956; Turner, 1970). The prominent non-arboreal pollen phase at the top of Stratum E is characteristic of a number of sites in the region (Turner, 1970; Thomas, 2001, 2002). Its origin is unclear, though it is not thought to show a cooling in climate (Turner, 1970).

### 4.4 Stratum D

This peat horizon indicates drying out of the lake basin and encroachment of terrestrial vegetation over the lake bed. The arboreal pollen contains significant quantities of alder, suggesting an alder carr environment developed during pollen zone HoIIIa. (West, 1956). Beetles indicate mean July temperatures of between 15 and 19 °C (Coope, 1993).

### 4.5 Stratum C

A return to lacustrine deposition is shown by the laminated sediments of Stratum C, which record fluctuating flows, with influx of coarser sands and silts, together with pellets of reworked lacustrine sediments and organic material. These were well exposed in Area VII. This stratum was originally assigned to pollen zone HoIIIb on the basis of the high counts of *Abies*, which is characteristic of this pollen zone (Turner, 1970). The occurrence of *Abies* and other thermophilous plants is, however, at odds with the presence of leaves of Arctic/Alpine plants, notably dwarf birch (*Betula nana*) and three species of dwarf willow (*Salix myrsinites*, *S. herbacea* and *S. polaris*) (Evans et al., 1896). Leaves of these species were recovered during the current work and are almost certainly contemporary with the unit, as they are fragile and would not survive reworking. This suggests that some of the pollen (including *Abies*) has been reworked into this unit (West, 1995) and furthermore indicates a hiatus between the deposition of Strata D and C.

The interpretation of a cold climate is also supported by the analysis of the beetles. Altogether, 72 coleoptran taxa have been recognised of which 42 can be named to species. Of these, 10 do not now live in the British Isles. There is little change through the sequence, so the species have been grouped together as a single assemblage in Table 2. The local environment suggests a pool of standing water with much marginal emergent vegetation such as the aquatic grass *Glyceria* and a surface which was at least in part covered with *Lemna*. The immediate surroundings of the pool were dominated by sedges and other reedy plants. The low numbers of dung beetles suggests that there were few large herbivorous mammals present at this time.

Taken as a whole, the coleopteran assemblage indicates very cold and continental climatic conditions with a number of species now found living today no nearer than arctic Russia (e.g. the closest locality for *Helophorus obscurellus* is on the Kanin Peninsula, the closest locality for *Holoboreaphilus nordenskioeldi* is central Novaya Zemlya). However, there are three species whose presence in this assemblage seems to be climatically anomalous. First, *Stenoscelis submuricatus* is a Mediterranean beetle that lives in dead wood. It was very common in Stratum D. This species could have been derived from Stratum D, having been incorporated into Stratum C (sealed from agents of decomposition inside reworked pieces of wood). On a less extreme scale, *Eledona agricola* lives in various fungi growing on deciduous trees, chiefly *Polyporus sulphuraeus* growing on *Salix*. In northern Europe, this beetle reaches only as far north as latitude 60°N. Species of *Throscus* inhabit leaf litter but again their geographical ranges only reach as far north as latitude 62°N. Both these records are based on single fragments and it is likely that they were also derived from the eroded deposits of the lacustrine sequence of Stratum D immediately beneath. Other than the probable derived elements, the insects indicate mean temperatures in July of, or below 10 °C and in January and February of about −15 °C.

### 4.6 Strata B2 and B1

The chalky sandy gravel of Stratum B2 is a fluvial sediment and contains a rich vertebrate fauna. The sands, silts and clays of Stratum B1 are also fluvial and rest in a channel cut into Stratum B2. The ‘Lower Industry’ in association with the vertebrate fauna was recovered along the northwest margins of this channel.

The vertebrate faunal assemblages from Strata B2 and B1 are very similar in composition (Stuart et al., 1993). The larger mammalian fauna is dominated by *Eguus. ferus* (horse), *Cervus elaphus* (red deer), *Dama dama* (fallow deer), together with occasional remains of *Macaca sylvanus* (macaque), *Ursus* sp. (bear), *Lutra lutra* (otter), *Panthera leo* (lion), *Stephanorhinus* sp. (extinct rhinoceros) and *Capreolus capreolus* (roe deer). Insectivores and rodents dominate the smaller mammals, which include *Castor fiber* (European beaver), *Trogontherium cuvieri* (extinct giant beaver), *Talpa minor* (extinct mole), *Microtus (Terricola)* cf. *subterraneus* (pine vole) and lemming (identified as *Lemmus lemmus* (Norway lemming) by Stuart et al., 1993). Remains of birds, amphibians, reptiles and fish were also recovered, including the articulated skeleton of a rudd (*Scardinius erythrophthalmus*) (Brian Irving, personal communication), the latter suggesting water temperatures were relatively warm during the summer months (cf. Stuart, 1982).

The range of species suggests a mix of environments. The dominance of horse indicates areas of open landscape, whereas forest is indicated by fallow deer, beaver and macaque. Although lemmings are only found in cold, northern latitudes today, they may have had a different distribution and habitat requirements in the Middle Pleistocene. For example, they occur at Boxgrove, West Sussex (Parfitt, 1999), during the latter part of an interglacial where they are associated with mammals and Mollusca typical of temperate deciduous woodland (e.g. *Myotis bechsteinii* (Bechstein's bat), *Muscardinus avellanarius* (common dormouse), *D. dama* (fallow deer), *Acanthinula aculeata*, *Spermodea lamellata* and *Aegopinella pura*).

### 4.7 Strata A2 and A1

The alluvial silt of Stratum A2(iii) contains a sparse fauna including an indeterminate species of elephant, extinct rhinoceros, horse, red deer, roe deer and fallow deer, again all suggesting a temperate climate. There is a possibility, however, that this fauna is derived from the lower units. The overlying sands and gravels of A2(ii), A2(i) and A1 contain no biological remains other than mixed pollen (Mullenders, 1993; Turner and West, 1994). Stratum A2(i) displays contemporaneous ice-wedge casts, indicating a permafrost environment (Singer et al., 1993). Periglacial structures in Stratum A1 also suggest a return to a cold climate.

### 4.8 Palaeoclimatic summary

The complete succession at Hoxne indicates a complex pattern of climatic fluctuations and changes in depositional regime. Following deglaciation, the lake basin began to infill under cool conditions (Stratum F) followed by rapid amelioration to full interglacial conditions which persisted throughout the accumulation of Stratum E. After a phase of non-lacustrine conditions when peat formed across the former lake basin (Stratum D), there is a hiatus and then a return to lacustrine conditions is indicated by Stratum C. By this time, climate had deteriorated with plant macrofossils and beetles indicating deposition under much colder conditions. In Area VII of the recent excavations, the top of Stratum C interdigitates with and is overlain by sand and fine, chalky gravel indicating increased flow into the basin and the establishment of a fluvial environment across the site (Stratum B2). This is incised by a further fluvial channel, which is infilled with fine-grained sediments (Stratum B1). The faunal elements within Stratum B suggest climatic amelioration, though probably not to the same extent as indicated by Strata E and D. Temperate climate also prevailed during deposition of Stratum A2(iii). The remainder of Strata A2 and A1 accumulated under cold climate conditions.

The archaeological assemblages of the Lower and Upper Industries and their associated mammalian assemblages can now be placed within this stratigraphic and environmental framework. No archaeological material can be securely attributed to Strata F–D. The Lower Industry is associated with the base of the channel-fill represented by Stratum B1 (Fig. 6). The Upper Industry was recovered from the upper part of Stratum A2(iii) and in a secondary context within overlying gravel, Stratum A2(ii). The critical consideration here is that both the Lower and Upper Industries can now be shown to post-date the ‘Arctic Bed’ of Stratum C.

The cold event represented by Stratum C and the temperate event represented by Stratum B have so far not been successfully dated or correlated with other terrestrial sequences or with the marine isotope record. Given the climatic complexity of MIS 11 (e.g. Bassinot et al., 1994; Tzedakis et al., 1997; Petit et al., 1999; Desprat et al., 2005) they could be correlated with later cold and warm events in MIS 11, or alternatively with even younger cold and warm episodes.

## 5 Amino acid geochronology

Amino acid racemization (AAR) analyses were undertaken on 12 *Bithynia tentaculata* opercula using the methods outlined in Penkman (2005) and Penkman et al. (2008). The method is based on the extent of protein decomposition, which increases with time, although there is an increased rate of breakdown during warm stages and a slowing in cold stages.

**Fig. 7:**
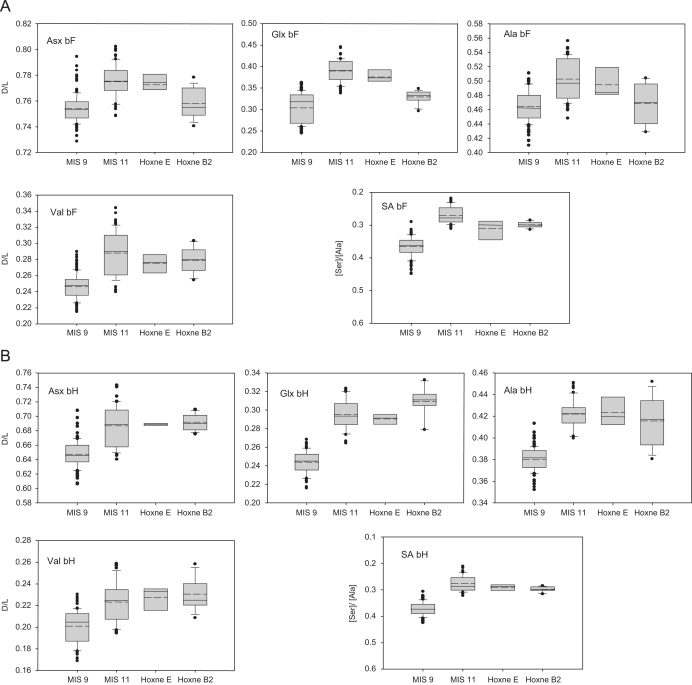
D/L values of Asx, Glx, Ala, Val and [Ser]/[Ala] for the (A) Free (FAA;F) and (B) Total Hydrolysable amino acid (THAA;H) fractions of bleached *Bithynia tentaculata* opercula from Hoxne (Strata E and B2), compared with shells from sites correlated with MIS 9 (Cudmore Grove, Grays, Hackney, Purfleet) and sites correlated with MIS 11 (Elveden, Ebbsfleet Southfleet Road, Swanscombe, Woodston, Clacton, Beeches Pit). For each group, the base of the box indicates the 25th percentile. Within the box, the solid line plots the median and the dashed line shows the mean. The top of the box indicates the 75th percentile. Where more than nine data points are available, the 10th and 90th percentiles can be calculated (shown by lines at the bottom and the top of the boxes, respectively). The results of each duplicate analysis are included in order to provide a statistically significant sample size. The y-axes for the [Ser]/[Ala] data are plotted in reverse, so that the direction of increased protein degradation for each of the indicators remains the same. Note: different scales on the *y*-axes.

The samples were from Stratum E (NEaar 0498–0500, 2446–2447) and Stratum B2 (NEaar 3143–3150). The results show levels of protein decomposition higher than those from sites correlated with MIS 9, but lower than those from sites of pre-Anglian age (Fig. 7; Table 3). Furthermore, the levels of protein decomposition are similar to those from sites correlated with MIS 11, including Elveden (Ashton et al., 2005), Beeches Pit (Preece et al., 2007), Barnham (Preece and Penkman, 2005), Clacton (Penkman et al., in press), Woodston and Swanscombe (Penkman, 2005). This indicates an age for Hoxne between the Anglian (MIS 12) and early MIS 9. The opercula samples from Stratum E tend to have slightly greater protein decomposition than those from Stratum B2 and less degraded protein than found in opercula from the Lower Freshwater Bed at Clacton, which was deposited early in MIS 11 (Bridgland et al., 1999).

The opercula from Stratum B2 have some of the lowest levels of protein decomposition determined from MIS 11 sites. This therefore suggests an age for the opercula between mid-MIS 11 and early MIS 9. While the values obtained from the opercula from Stratum B2 generally show higher levels of protein decomposition than those obtained from the MIS 9 sites analysed, the separation between the Stratum B2 samples and those deposited early within MIS 9 is small. As so little decomposition occurs in the cold stages, and because of the extent of natural variability in biological samples, it can be difficult to discriminate the end of one warm stage from the beginning of the next. Although an age late in MIS 11 is more likely, it is not possible to rule out an early MIS 9 age, given the level of resolution currently obtainable from the technique.

## 6 Biostratigraphy

The mammalian fauna from Strata B1 and B2 also provides an indication of age. Three species are of possible biostratigraphic significance. The most important of these is *Microtus (Terricola)* cf. *subterraneus*. Although it is widespread in Europe today, it appears to have been absent in Britain after MIS 11 (Parfitt, 1998). Of particular significance is its absence from the very rich faunal assemblages from Cudmore Grove, Grays and Purfleet, all of which have been attributed to MIS 9 and from any younger sites (Bridgland, 1994; Schreve et al., 2002).

Of lesser significance is the presence of *Trongontherium cuvieri* and *Talpa minor*. Although they are thought to have become extinct after the Hoxnian in Britain, and possibly the Holsteinian in Europe, their remains are so rare that any apparent absence in sites attributed to MIS 9 or later might be due to insufficient sampling.

## 7 Discussion

Both the amino acid geochronology and the biostratigraphy, together with the reassessment of the ESR dates (Grün and Schwarcz, 2000; see above), suggest that Strata B1 and B2 are most likely to be attributable to MIS 11. This therefore implies that the underlying Strata E and D (Hoxnian) date to the first prolonged temperate substage in MIS 11, and that Strata C and B are later cold and warm substages, respectively, within MIS 11.

This correlation of the sequence at Hoxne with substages of MIS 11 has wider implications for its correlation with other terrestrial sites in the UK and further afield. Although the full Hoxnian Interglacial sequence is not found at Hoxne, a complete succession is found at Marks Tey (Fig. 2), where pollen zones HoI-IV are represented (Turner, 1970). Furthermore, the palynology suggests that there is no evidence for a hiatus between the Anglian till and the lacustrine sediments at either Hoxne or Marks Tey. The Hoxnian record at Marks Tey is an overlapping composite sequence from two main cores. The interpretation of these cores is of a continuous temperate sequence through the Hoxnian without any indication of a cold event. Together with the evidence from Hoxne of a later MIS 11 cold substage, this suggests that the Hoxnian Interglacial can be equated with the first major temperate substage within MIS 11.

**Fig. 8:**
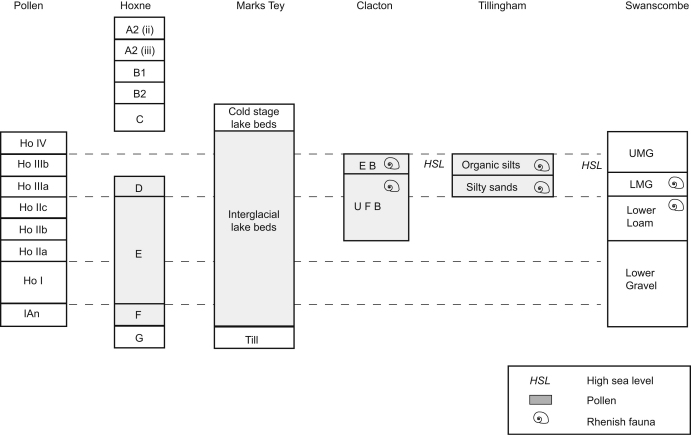
Suggested correlation between Hoxne, Marks Tey, Clacton, Tillingham and Swanscombe with pollen zones. For Clacton, UFB=Upper Freshwater Bed and EB=Estuarine Bed. For Swanscombe, LMG=Lower Middle Gravel and UMG=Upper Middle Gravel.

Palynology has also been used to correlate the lacustrine deposits at Hoxne and Marks Tey with the organic channel-fills at Clacton and Tillingham, which form part of the Thames/Medway sequence (Fig. 2). At Clacton, the Freshwater Beds and Estuarine Bed (Pike and Godwin, 1953) have been correlated with HoIIb–HoIIIb (Kerney, 1971; Bridgland et al., 1999), while at Tillingham, the silty sands and organic silts are attributed to HoIII (Roe, 2001) (Fig. 8). On the basis of their lithology, terrace stratigraphy and molluscan assemblages (Bridgland, 1994; Roe, 2001; Preece et al., 2007), both these sites are argued to be part of the same terrace aggradation as the Lower Gravel, Lower Loam and Middle Gravels at Swanscombe (Fig. 2). All three sites record the immigration of the ‘Rhenish’ fauna, probably in late HoII (indicating a confluence of the Thames with the Rhine). Furthermore, the presence of estuarine molluscs indicates a high sea-level stand, argued from the evidence at Clacton and Tillingham to occur during HoIIIb (Fig. 8).

These correlations therefore suggest that the sequences at Clacton, Tillingham and the Lower Gravel to Middle Gravels at Swanscombe can also be attributed to the first temperate substage of MIS 11. This is at variance with the interpretation of Swanscombe proposed by Schreve (2001a, b) who attributed the Middle Gravels to a later temperate substage within MIS 11.

The interglacial sequence at Quinton in the West Midlands has been interpreted as spanning the entire Hoxnian Interglacial on the basis of its palynology (Horton, 1989). However, evidence from the Coleoptera indicates a more complex climatic picture with a ‘cold interlude’ occurring during the latter part of the interglacial (Coope and Kenward, 2007). The beetle fauna from this ‘cold interlude’ is very similar to that of the ‘Arctic Bed’ at Hoxne and suggests a possible correlation. Alternatively, since the uppermost samples of the Quinton sequence, attributed to the onset of the succeeding glacial, also yielded a similar suite of cold-adapted beetle species, correlation of the Hoxne Arctic Bed with these uppermost samples at Quinton is a possibility. The beetle evidence for the ‘cold interlude’ at Quinton is at odds with the palynological evidence which “does not appear to show any response to this cold episode” (p. 3284). A similar discrepancy in the evidence from Stratum C at Hoxne has been accounted for by reworking of temperate pollen into Stratum C (see above and Turner, 1970). A comparable situation may have occurred at Quinton where pollen of temperate character is reworked from the underlying deposits and found in conjunction with an autochthonous coleopteran assemblage indicative of cooler climatic conditions. A re-evaluation of the palynology of the Quinton succession may help to resolve these problems associated with correlation of the Hoxne and Quinton sequences.

**Fig. 9:**
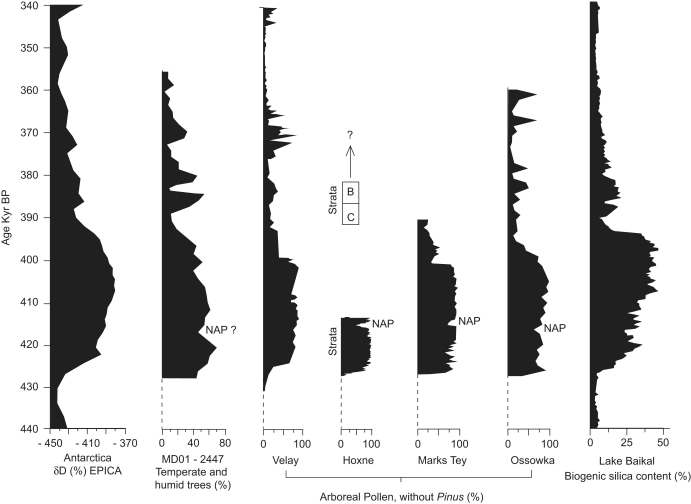
Correlation of MIS 11 sites across Eurasia with the Antarctic ice core based on data derived from EPICA Community members (2004), Desprat et al. (2005), Reille and de Beaulieu (1995), West (1956), Turner (1970), Nitychoruk et al. (2005) and Prokopenko et al. (2001). The arboreal pollen (AP) curves for Velay, Hoxne, Marks Tey and Ossowka do not include pine. The EPICA, MD01–2447, Velay and Lake Baikal records are plotted using the timescales published in the original reports. The AP curves from Hoxne, Marks Tey and Ossowka have been converted to a timescale by correlation with the Velay and MD01–2447 records based on three tie-points: the rapid increase of AP at the end of the Anglian/Elsterian glaciation; the NAP phase midway through the Hoxnian/Holsteinian Interglacial; and the sudden decrease in AP at the end of the interglacials.

In Europe, significant advances have been made over the last decade in relating the vegetational record from long, continuous sequences from sites in southern Europe to the marine isotope record. Key to this success has been core MD01–2447, near the northwest coast of the Iberian Peninsular (Fig. 2), where the marine isotope record can be directly compared to pollen that reflects vegetational changes inland (Desprat et al., 2005). This core is argued to span the last 426 ka and has been compared to other continuous or composite palynological sequences from Tenaghi Philippon in Greece, and Velay maar sites (Praclaux, Le Bouchet and Ribains; Fig. 2) in France (Reille and de Beaulieu, 1995; Tzedakis et al., 1997, 2001, 2006). All these sequences show a similar pattern of vegetation and climate change with successive interglacial/glacial cycles. These can be related to records of global climate change from deep-sea cores (Oppo et al., 1998; McManus et al., 1999), ice cores (Petit et al., 1999; EPICA Community Members, 2004) and the changes in the biogenic silica content in the sequence from Lake Baikal (Prokopenko et al., 2001) (Fig. 9). Absolute dates from the terrestrial sites support these correlations with ^40^Ar/^39^Ar dates on trachytic tephra in deposits of the third interglacial at Velay (Le Bouchet Interglacial) suggesting an MIS 7 age (de Beaulieu et al., 2001), and palaeomagnetic analyses and U-series dates on the sequence at Tenaghi Philippon providing further tie-points to the marine isotope record (Tzedakis et al., 1997, 2006).

For core MD01–2447, the first part of MIS 11 has been characterised as showing a long, marked warm period from 426 to 394 ka called the Vigo Interglacial, which has been correlated with the Praclaux Interglacial at the Velay sites. The Praclaux Interglacial has also been argued to be similar to the Holsteinian palynological records of northern Europe (Reille and de Beaulieu, 1995; Turner, 1998; de Beaulieu et al., 2001; Desprat et al., 2005; though also see Geyh and Müller, 2005; Geyh and Müller, 2006; Scourse, 2006) and to the Hoxnian Interglacial (Turner, 1998). The evidence from Hoxne would support this interpretation. Key features of the Hoxnian pollen records are the early occurrence of *Picea*, the development of mixed oak forest followed by a marked phase of *Abies*, and the occurrence of *Pterocarya* in the later part of the interglacial. We therefore conclude that the Hoxnian, Holsteinian, Vigo and Praclaux interglacials all correlate with the first part of MIS 11.

In the later part of MIS 11, three cold/warm cycles have been recognised in core MD01–2447. These cycles are similar to a series of short-lived cold/warm phases in the Velay sites, which occur after the Praclaux Interglacial and prior to the Bargette cold episode of MIS 10. At Velay, two stadial/interstadial cycles have been named (Chaconac stadial/Jagonas 1 interstadial and Coucouron stadial/Jagonas 2 interstadial).

During the stadials, the pollen from core MD01–2447 indicates that either heath or dry grassland dominated the local vegetation, while at Praclaux the environment was open with an abundance of steppe taxa. The interstadials indicate the re-emergence of forest cover with some deciduous woodland. In core MD01–2447, *Pinus* and *Quercus* are prominent, with lesser quantities of *Carpinus* and *Abies*. Pine, however, was argued to be over-represented due to better dispersal ability and buoyancy. The upland site of Praclaux is characterised during these interstadials by the dominance of *Picea*, but also by the presence of *Carpinus*, *Quercus*, *Buxus*, *Fraxinus* and *Tilia*. It is suggested that the presence of *Carpinus* (up to 10%) may indicate that there was a greater abundance of this taxon at lower altitudes (Reille and de Beaulieu, 1995).

How far north this deciduous woodland stretched is difficult to gauge, due to the paucity of sites that clearly correlate with these phases. Although there is no unequivocal palaeobotanical information on the vegetation at Hoxne from Stratum B, the mammalian fauna includes obligate woodland species (e.g. beaver, fallow deer and macaque) providing strong evidence that there must have been some forest cover.

The evidence from Hoxne, therefore, suggests that the ‘Arctic Bed’ of Stratum C and the temperate phase of Stratum B correlate with one of the cold/warm cycles in the later part of MIS 11, although because of the hiatus between Stratum D and C, it is not clear to which cycle they should be attributed. The problem of recycled pollen in both Strata C and B also makes it difficult to reconstruct their vegetation histories, other than the survival of leaves of dwarf birch and dwarf willow, in Stratum C. However, core MD01–2447 and Praclaux provide clues about the vegetation that might have been present at Hoxne, despite differences in latitude, and in the case of Praclaux in altitude (1100 m, compared to Hoxne at 30 m) between the sites.

Elsewhere in northern Europe, there is little agreement on the correlation of post-Holsteinian temperate events. Most authorities would now agree that the Holsteinian is attributable to MIS 11. If the interpretation favoured here is correct, that the Hoxnian and Holsteinian both correlate with the first temperate event of MIS 11, then this still leaves the question of whether later MIS 11 interstadials can be recognised in northern Europe.

One of the best Holsteinian pollen records comes from the lacustrine sequence at Ossowka in eastern Poland (Nitychoruk et al., 2005; Fig. 2). This sequence has been constrained by TL dates of ca 430 ka at the MIS 12/11 boundary, and the estimation of the duration of the sequence is calculated from annual laminations in the interglacial part of the record. Like the pollen sequences in southern Europe, after a stable temperate climate of an estimated 35–39 ka (the Holsteinian), there follows a series of climatic oscillations with open, cold vegetation alternating with a boreal environment dominated by pine. If the estimated timescale of Nitychoruk et al. (2005) is correct, this would imply that the later temperate events in MIS 11 are characterised by boreal pine forest in central, northern Europe.

Correlation with other north European sites (e.g. Bilzingsleben and Schöningen) is as yet uncertain due to the varying interpretations that are currently put forward (cf. Mania, 1995; Urban, 1995, 2007; Turner, 1998; Bridgland et al., 2006). However, it is worth noting some of the similarities between the Channel II, Level 4b deposits at Schöningen to Stratum B at Hoxne. Level 4b, which includes most of the spears, is assigned to the Reinsdorf B Interstadial (Kolfschoten, 1993). The fauna is dominated by horse and the pollen indicates boreal forest predominantly of pine, but with some spruce, birch and larch (Urban, 2007).

The differences in the vegetational records from southern to northern Europe would suggest quite a marked climatic gradient between 40° and 50° latitude during the later interstadials of MIS 11. A similar pattern has also been identified for MIS 5, where the vegetational records for substages 5c and 5a at Grande Pile (France; Woillard, 1978) show deciduous woodland, whereas those further north in the Netherlands, Germany, Denmark and, to a lesser extent, the UK show that the vegetation was dominated by boreal forest (Behre, 1989; Turner, 1998). Turner suggests that either the phases were too short to allow for the immigration of thermophilous trees, or that there was a real climatic barrier to the spread of deciduous woodland to the north. This may be related to circulation patterns in the North Atlantic Ocean, with a southerly shift in the Gulf Stream, leading to even cooler temperatures in northern Europe. There was also likely to have been a west–east gradient in climate; Zagwijn (1990) has suggested that summer temperatures during substage 5c showed a marked decrease from the southwest to the northeast in Europe, unlike substage 5e, where the gradient was from southeast to northwest. Although during substage 5c there seem to be few vegetational differences between sites in Britain and those further east, where forests of pine, birch and occasionally spruce were dominant (Behre, 1989), it has been suggested that Britain had a more continental climate with cold winters, but warm summers (Coope, 1977). If this can be used as an analogue for the late MIS 11 interstadials, then Hoxne might have had vegetation of boreal forest, but with warm summer temperatures. This conclusion is supported by the faunal evidence from Stratum B at Hoxne.

## 8 Conclusions

Hoxne is a key site for understanding the Middle Pleistocene sequence of northern Europe and understanding how this correlates with sequences from southern Europe. The site provides a stratigraphic sequence that includes two post-Anglian temperate phases. The first of these (the Hoxnian) is argued to correlate with the first sustained temperate phase in MIS 11 between ca 425–395 ka. The second, as represented by Stratum B, is correlated with a later interstadial in MIS 11. These two temperate phases may be tentatively correlated with substages 11c and 11a, respectively, which are evident in the SPECMAP stack (Imbrie et al., 1984; Tzedakis et al., 2001). Alternatively, the acme of the Hoxnian may be correlated with isotopic event 11.3 and Stratum B with either event 11.23 or 11.1 of Bassinot et al. (1994). The intervening cold episode, represented by Stratum C is correlated with marine isotope substage 11b and may equate to either event 11.24 or 11.22 in the Low Latitude Stack (Bassinot et al., 1994).

Lithostratigraphy, palynology and molluscan data suggest that the sequences at Clacton, Tillingham and the Lower Gravels to Middle Gravels at Swanscombe can be attributed to the first temperate event (the Hoxnian). Comparison with the continuous palynological records from southern Europe suggests that the Hoxnian correlates with the Vigo Interglacial of northwest Iberia, the Praclaux Interglacial of the Velay maars sites and to the Holsteinian Interglacial of northern Europe. Stratum B is argued to correlate with either the Jagonas 1 or 2 Interstadial from the Velay sites. Reconstruction of the vegetation during these interstadials suggests that in northern Europe they were dominated by a pine-birch boreal forest, which supported a diverse large mammal fauna.

Traditionally, these faunas have been interpreted as indicating fully interglacial conditions. The evidence from Hoxne, therefore, clearly indicates that similar faunal assemblages can also occur in environments of interstadial character. This has implications for the biostratigraphical subdivision of temperate episodes in the Middle Pleistocene based on mammalian evidence (cf. Schreve, 2001a, b).

Hoxne is also an important site for understanding the Lower Palaeolithic occupation of northern Europe. The archaeological assemblages at Hoxne can now be shown to date to an interstadial that has not been previously recognised in Britain. Although there are several MIS 11 sites where fine-grained, organic sediments allow detailed environmental reconstruction, they have all suggested that human occupation was associated with deciduous woodland in fully temperate climate (cf. Ashton et al., 2006). At Hoxne, however, humans can be demonstrated to have lived in a boreal forest environment and probably with distinctly cooler winters. This prompts questions about the technologies required (clothing, shelters, control of fire) or physical adaptations needed in order to survive these cooler environments. The range of environments that humans inhabited during the Middle Pleistocene has long been the subject of debate (Gamble, 1987, 1992; Roebroeks et al., 1992). Hoxne now adds to the small list of sites from the Lower Palaeolithic where the human habitat can be reconstructed in more detail and indicates human adaptability to a range of different habitats.

## Figures and Tables

**Table 1:** New bed names and descriptions with interpretation of climate and context of archaeology

Bed name	Bed description	Pollen zone	Climatic interpretation	Archaeology
Stratum A1	Coversand		Cold	
Stratum A2(i)	Cryoturbated sand and gravel		Cold	
Stratum A2(ii)	Solifluction gravel		Cold	Derived Upper Industry
Stratum A2(iii)	Alluvial sandy clay		Warm	Upper Industry
Stratum B1	Fluvial sand, silt and clay		Warm	Lower Industry
Stratum B2	Fluvial chalky gravel		Warm	
Stratum C	Lacustrine sands and silts		Cold	
*Hiatus*				
Stratum D	Peat	HoIIIa	Warm	
Stratum E	Lacustrine clay	HoI–IIc	Warm	
Stratum F	Lacustrine clay	lAn	Cool	
Stratum G	Till		Cold	

**Table 2:** Coleoptera from Stratum C, Hoxne

Carabidae	
*Notiophilus* cf. *aquaticus* (L.)	2
*Dyschirius globosus* (Hbst.)	3
*Trechus secalis* (Payk.)	1
*Bembidion hasti* Sahlb.^a^	1
*Bembidion* cf. *mckinleyi* Fall.^a^	1
*Bembidion guttula* (F.)/*unicolor* Chaud.	2
*Bembidion* sp.	4
*Patrobus* cf *atrorufus* (Ström)	2
*Pterostichus nigrita* (Payk.)	1
*Amara* sp.	2
Dytiscidae	
*Potamonectes griseostriatus* (de Geer)	3
*Agabus bipustulatus* (L.)	1
*Ilybius* sp.	2
*Rhantus* sp.	1
*Colymbetes dolabratus* (Payk.)^a^	2
*Colymbetes* sp.	5
*Graphoderus* sp.	1
*Dytiscus* sp.	1
Gyrinidae	
*Gyrinus aeratus* Steph.	1
*Gyrinus* sp.	2
Hydraenidae	
*Hydraena* sp.	5
*Ochthebius minimus* (F.)	7
*Helophorus obscurellus* Popp.^a^	6
*Helophorus* cf. *aquaticus* (L.)	1
*Helophorus* small spp.	5
Hydrophilidae	
*Cercyon convexiusculus* Steph.	4
*Enochrus* sp.	2
*Hydrobius fuscipes* (L.)	7
Orthoperidae	
*Orthoperus* sp.	2
Ptiliidae	
*Ptenidium* sp.	2
*Acrotrichis* sp.	2
Staphylinidae	
*Pycnoglypta lurida* (Gyll.)^a^	1
*Olophrum fuscum* (Grav.)	7
*Olophrum boreale* (Payk.)	2
*Eucnecosum brachypterum* (Grav.)	24
*Geodromicus kunzei* Heer	1
*Boreaphilus henningianus* Sahlb.^a^	5
*Holoboreaphilus nordenskioeldi* (Makl.)^a^	6
*Trogophloeus* sp.	4
*Oxytelus rugosus* (F.)	1
*Bledius* sp.	1
*Stenus* spp.	7
*Euaesthetus laeviusculus* Mannh.	1
*Lathrobium* sp.	1
*Tachyporus* sp.	1
*Tachinus rufipes* (de Geer)	1
*Tachinus* cf. *corticinus* Grav.	1
Alaeocharinae gen. et sp. indet.	59
Elateridae	
*Agriotes* sp.	1
Throscidae	
*Throscus* sp.	1
Helodidae	
gen. et sp. indet.	7
Dryopidae	
*Dryops* sp.	3
Byrrhidae	
*Simplocaria metallica* (Sturm.)^a^	6
Coccinellidae	
*Hippodamia arctica*^a^	1
Tenebrionidae	
*Eledona agaricola* (Hbst.)	1
Scarabaeidae	
*Aphodius* spp.	2
Chrysomelidae	
Donacia dentata Hoppe	2
*Donacia semicuprea* Panz.	5
*Donacia aquatica* (L.)	1
*Donacia thalassina* Germ.	9
*Donacia cinerea* Hbst.	2
*Plateumaris affinis* (Kunze)	4
*Chrysomela* sp.	1
Curculionidae	
*Apion* sp.	1
*Sitona* sp.	1
*Stenoscelis* (=*Brachytemnus*) *submuricatus* (Schönh.)^a^	3
*Bagous* sp.	2
*Tanysphyrus lemnae* (Payk.)	9
*Notaris bimaculatus* (F.)	4
*Notaris acridulus* (L.)	1
*Notaris aethiops* (F.)	2
*Thryogenes* sp.	1

a Indicates species not now native to the British Isles.

**Table 3:** Amino acid data on opercula of *Bithynia tentaculata* from Strata E and B2 at Hoxne

NEaar no.	Sample name	Asx D/L	Glx D/L	Ser D/L	Ala D/L	Val D/L	[Ser]/[Ala]
0498bF	HoBto1bF	0.769±0.000	0.366±0.001	0.745±0.000	0.480±0.003	0.261±0.000	0.359±0.001
0498bH*	HoBto1bH*	0.686±0.001	0.293±0.000	0.743±0.002	0.424±0.002	0.236±0.002	0.308±0.001
0500bF	HoBto2bF	0.782±0.001	0.369±0.029	0.990±0.002	0.531±0.001	0.285±0.008	0.283±0.004
0500bH*	HoBto2bH*	0.692±0.002	0.296±0.001	0.750±0.004	0.444±0.003	0.235±0.002	0.281±0.003
2446bF	HoBto3bF	0.777±0.002	0.374±0.000	1.051±0.006	0.483±0.003	0.271±0.003	0.302±0.002
2446bH*	HoBto3bH*	0.689±0.002	0.283±0.001	0.754±0.006	0.413±0.005	0.216±0.002	0.287±0.000
2447bF	HoBto4bF	0.763±0.014	0.395±0.002	1.044±0.005	0.485±0.003	0.285±0.003	0.296±0.004
2447bH*	HoBto4bH*	0.689±0.000	0.290±0.000	0.748±0.013	0.412±0.001	0.223±0.014	0.282±0.008
3143bF	Ho64Bto1bF	0.745±0.006	0.332±0.003	1.028±0.004	0.434±0.008	0.256±0.002	0.310±0.006
3143bH*	Ho64Bto1bH*	0.689±0.001	0.305±0.000	0.805±0.013	0.390±0.000	0.220±0.006	0.310±0.002
3144bF	Ho64Bto2bF	0.752±0.004	0.346±0.004	1.030±0.007	0.457±0.002	0.287±0.001	0.300±0.003
3144bH*	Ho64Bto2bH*	0.690±0.002	0.311±0.000	0.807±0.001	0.395±0.001	0.223±0.003	0.315±0.001
3145bF	Ho64Bto3bF	0.775±0.005	0.334±0.006	1.037±0.001	0.498±0.007	0.286±0.003	0.286±0.002
3145bH*	Ho64Bto3bH*	0.696±0.002	0.311±0.000	0.783±0.009	0.435±0.000	0.236±0.002	0.289±0.001
3146bF	Ho64Bto4bF	0.749±0.001	0.300±0.005	1.013±0.003	0.440±0.001	0.269±0.000	0.306±0.000
3146bH*	Ho64Bto4bH*	0.680±0.002	0.279±0.000	0.764±0.001	0.383±0.003	0.210±0.002	0.300±0.000
3147bF	Ho50Bto1bF	0.748±0.005	0.342±0.002	1.028±0.011	0.439±0.015	0.264±0.003	0.298±0.008
3147bH*	Ho50Bto1bH*	0.709±0.001	0.332±0.001	0.833±0.000	0.415±0.000	0.240±0.001	0.293±0.009
3148bF	Ho50Bto2bF	0.767±0.002	0.337±0.005	1.012±0.032	0.503±0.001	0.303±0.001	0.289±0.003
3148bH*	Ho50Bto2bH*	0.703±0.003	0.321±0.001	0.784±0.000	0.450±0.003	0.257±0.002	0.286±0.000
3149bF	Ho50Bto3bF	0.759±0.006	0.327±0.007	1.026±0.000	0.482±0.002	0.275±0.001	0.297±0.001
3149bH*	Ho50Bto3bH*	0.679±0.003	0.306±0.003	0.754±0.011	0.419±0.004	0.223±0.003	0.298±0.001
3150bF	Ho50Bto4bF	0.771±0.000	0.321±0.002	0.771±0.001	0.496±0.000	0.294±0.001	0.310±0.001
3150bH*	Ho50Bto4bH*	0.701±0.003	0.312±0.006	0.686±0.005	0.437±0.005	0.242±0.003	0.286±0.002

Error terms represent 1 S.D. about the mean for the duplicate analyses for an individual sample. Each sample was bleached (b), with the free amino acid fraction signified by ‘F’ and the total hydrolysable fraction by ‘H*’. NEaar 0498-0500 and 2446-2447 are from Stratum E, and NEaar 3143-3150 are from Stratum B2.
